# ﻿The identity of *Bupleurumjeholense* (Apiaceae)

**DOI:** 10.3897/phytokeys.237.111977

**Published:** 2024-01-31

**Authors:** Li-Hua Wang, Xue-Min Xu, Yi He, Quan-Ru Liu

**Affiliations:** 1 Key Laboratory of Biodiversity Science and Ecological Engineering, Ministry of Education, College of Life Sciences, Beijing Normal University, Beijing 100875, China Beijing Normal University Beijing China; 2 School of Life Sciences, Zhengzhou University, Zhengzhou 450001, Henan China Zhengzhou University Zhengzhou China

**Keywords:** Apiaceae, *Bupleurumchinense* DC. var. *jeholense* (Nakai) Q.R.Liu & L.H.Wang, new combination

## Abstract

*Bupleurumjeholense* Nakai (Apioideae, Apiaceae), originally found in the Wuling Mountain of China, was initially described as a species but later treated as a variety of *B.sibiricum* Vest ex Spreng. However, researchers have recently found that it is more closely related to *B.chinense* DC. In this study, we conducted morphological and phylogenetic analyses as well as chromosome counting to determine the taxonomic status of B.sibiricumvar.jeholense (Nakai) Chu. Our results showed that B.sibiricumvar.jeholense and *B.chinense* share common features (i.e., bracteoles 5 and stem solid) that distinguish both from B.sibiricumvar.sibiricum. The chromosome number of B.sibiricumvar.jeholense was found to be the same as in *B.chinense* (i.e., 2n = 12), whereas the chromosome number of B.sibiricumvar.sibiricum was 64. A phylogenetic tree based on complete chloroplast genome data revealed a close relationship between B.sibiricumvar.jeholense and *B.chinense*. Finally, B.sibiricumvar.jeholense and *B.chinense* were mainly found to differ in plant height, number of stems, and middle stem leaves. Based on this evidence, we propose a new combination: Bupleurumchinensevar.jeholense (Nakai) Q.R.Liu & L.H.Wang.

## ﻿Introduction

*Bupleurum* is a monophyletic genus in the tribe Bupleureae (Apioideae, Apiaceae) (Downie et al. 2000). It contains around 180–195 species, is distributed throughout Eurasia and North Africa, with one species each in North America and South Africa, while being adventive in Australasia (Plunkett et al. 2019). China is a major diversity center of *Bupleurum*, containing 42 species and 16 varieties (Sheh and Watson 2005). This genus can be easily recognized based on its entire and single leaves, which usually show parallel venation and distinct bracts and bracteoles. However, the morphological characteristics used for species delimitation of this genus remain limited, thereby rendering species identification difficult. To address this problem, it is necessary to perform detailed examinations and observations in field populations.

*Bupleurumjeholense* Nakai was initially described on the basis of specimens collected from the Wuling Mountain (China). Nakai stated that the characters of this species are as follows: middle stem leaves oblanceolate, base tapering, apex obtuse or acute, apiculate, bracteoles 5, exceeding flowers (Nakai 1937). Later, Chu (Shan and Li 1974) treated *B.jeholense* as a variety of *B.sibiricum* Vest ex Spreng., i.e., B.sibiricumvar.jeholense (Nakai) Chu. Chu reported that *B.jeholense* was similar to *B.sibiricum*, with the main difference being in bracteole number. For example, *B.jeholense* commonly has five bracteoles, whereas *B.sibiricum* usually has 7–12 bracteoles. This difference was thought to be associated with a geographic distribution, i.e., the transition from the main distribution area in Siberia to the limited populations found in the Wuling Mountain (Liaoning Forestry Soil Research Institute 1977).

During recent fieldwork on Dongling Mountain (Beijing, China), we found that the distribution of *Bupleurum* species was closely linked to altitude. Normally, *B.chinense* is distributed at low altitudes (<1600 m). However, with the increasing altitude, we found a continuous variation in *B.chinense*; for example, plants became shorter; the number of branches decreased; the number of bracteoles gradually changed from 3 to 5 and varied in length from obviously shorter than the umbel to almost equal. Furthermore, as the altitude increased to 1600 m, *B.chinense* was gradually replaced by B.sibiricumvar.jeholense. After checking the specimens, we also found that there were misidentifications between *B.chinense* and B.sibiricumvar.jeholense. Subsequent phylogenetic analyses of Chinese *Bupleurum* spp. based on nrDNA ITS and chloroplast markers (i.e., *trnH-psbA* and *matK*) indicated that B.sibiricumvar.jeholense was more closely related to *B.chinense*, whereas B.sibiricumvar.sibiricum was closely related to *B.smithii* Wolff (Wang et al. 2011b). Hence, we doubt whether B.sibiricumvar.jeholense is a morphological variation of *B.chinense* that is adapted to high-altitude locations in the Yan Mountains.

We therefore collected and checked several specimens from the type locality of *B.jeholense*. We conducted morphological observations, statistical comparisons, cytological studies, and a phylogenetic analysis based on the complete chloroplast genome to clarify the relationship among B.sibiricumvar.jeholense, B.sibiricumvar.sibiricum, and *B.chinense*.

## ﻿Materials and methods

### ﻿Morphological observations

Using the existing scientific literature and the relevant type specimens, we collected new specimens from the type locality of B.sibiricumvar.jeholense and compared these with images of the type specimens. Specimens from field collections, CVH (https://www.cvh.ac.cn/), and online herbarium collections (MW, LE) were used to count bracteoles. In total, we obtained bracteole count data for 129 specimens of B.sibiricumvar.jeholense, 183 specimens of B.sibiricumvar.sibiricum, and 183 specimens of *B.chinense*. We analyzed these data using R (beanplot package) to produce boxplots (Kampstra 2008). Morphological terminology was used according to Kljuykov et al. (2004). Voucher specimens were deposited to the BNU herbarium.

### ﻿Cytology

All materials used for cytological studies were obtained from the Wuling Mountain (B.sibiricumvar.jeholense), Wutai Mountain (*B.chinense*), and Daqing Mountain (B.sibiricumvar.sibiricum). All voucher specimens are listed in Table 1. Chromosome preparations were produced using acid digestion and hypotonic wall removal and photographed. Three technical replicates were obtained for each taxon. This procedure was adapted from Li et al. (2021).

**Table 1.** Voucher information and GenBank accession numbers for newly sequenced plastome sequences.

Taxon	Location	Voucher information	Accession
*B.chinense*	Dongling Mountain, Beijing, China	BNU2021HB002 (BNU)	OR387523
*B.smithii*	Xiaowutai Mountain, Hebei, China	BNU2020DT007(BNU)	OR387522
B.sibiricumvar.sibiricum	Daqing Mountain, Inner Mongolia, China	BNU2021NMG017(BNU)	OR387525
B.sibiricumvar.jeholense	Wuling Mountain, Hebei, China	BNU2021HB025(BNU)	OR387524

### ﻿Sampling and molecular analysis

Fresh plant leaves were collected from the field and quickly dried with silica gel for DNA extraction. Specimen voucher information is shown in Table 1. DNA was extracted using an HP Plant DNA hypotonic (D2485-02; Omega Bio-Tek). DNA samples were then sent to Beijing Novogene Corporation for quality testing and resequencing. An Illumina HiSeq X sequencing platform was used to generate approximately 10 GB of data for each sample. The chloroplast genome was then assembled from clean data using GetOrganelle (Jin et al. 2020). PGA (Qu et al. 2019) was used to annotate the resulting chloroplast genome. Sequences for *B.yinchowense* Shan et Y.Li (MT075711) and *B.sikangense* X.J.He & C.B.Wang (NC056803) were downloaded from NCBI (https://www.ncbi.nlm.nih.gov/nucleotide/) to be used as references. All chloroplast genome sequences generated here were deposited in the NCBI GenBank database (accession numbers listed in Table 1). Finally, twenty-one plastid genome sequences were downloaded from NCBI (see Appendix) for phylogenetic comparisons. This included 19 species of *Bupleurum* and two species of *Pleurospermum* Hoffm., which were used as outgroups.

In total, 25 sequences were imported into PhyloSuite (Zhang et al. 2020). The mafft module (Katoh et al. 2019) was used for sequence alignment, and the ModelFinder module (Kalyaanamoorthy et al. 2017) was used to calculate the nucleotide substitution model for all aligned sequences. A maximum likelihood (ML) tree was then constructed using IQ-TREE (Minh et al. 2020), with the nucleotide substitution model set to TVM+F+R2 and a standard bootstrap value of 1000. Results were considered reliable when the bootstrap support value (BS) was ≥70% (Kress et al. 2002). A Bayesian (BI) tree was constructed using MrBayes (Huelsenbeck and Ronquist 2001), with the GTR+F+I+G4 model using the following settings: mcmcp ngen = 2,000,000, printfreq = 10,000, nchains = 4, and burninfrac = 25%. Results were considered reliable when the posterior probability (PP) was ≥0.95. The effective sample size (>200) was determined using Tracer version 1.7 (Rambaut et al. 2018).

## ﻿Results

### ﻿Morphological observations

**Figure 1.**
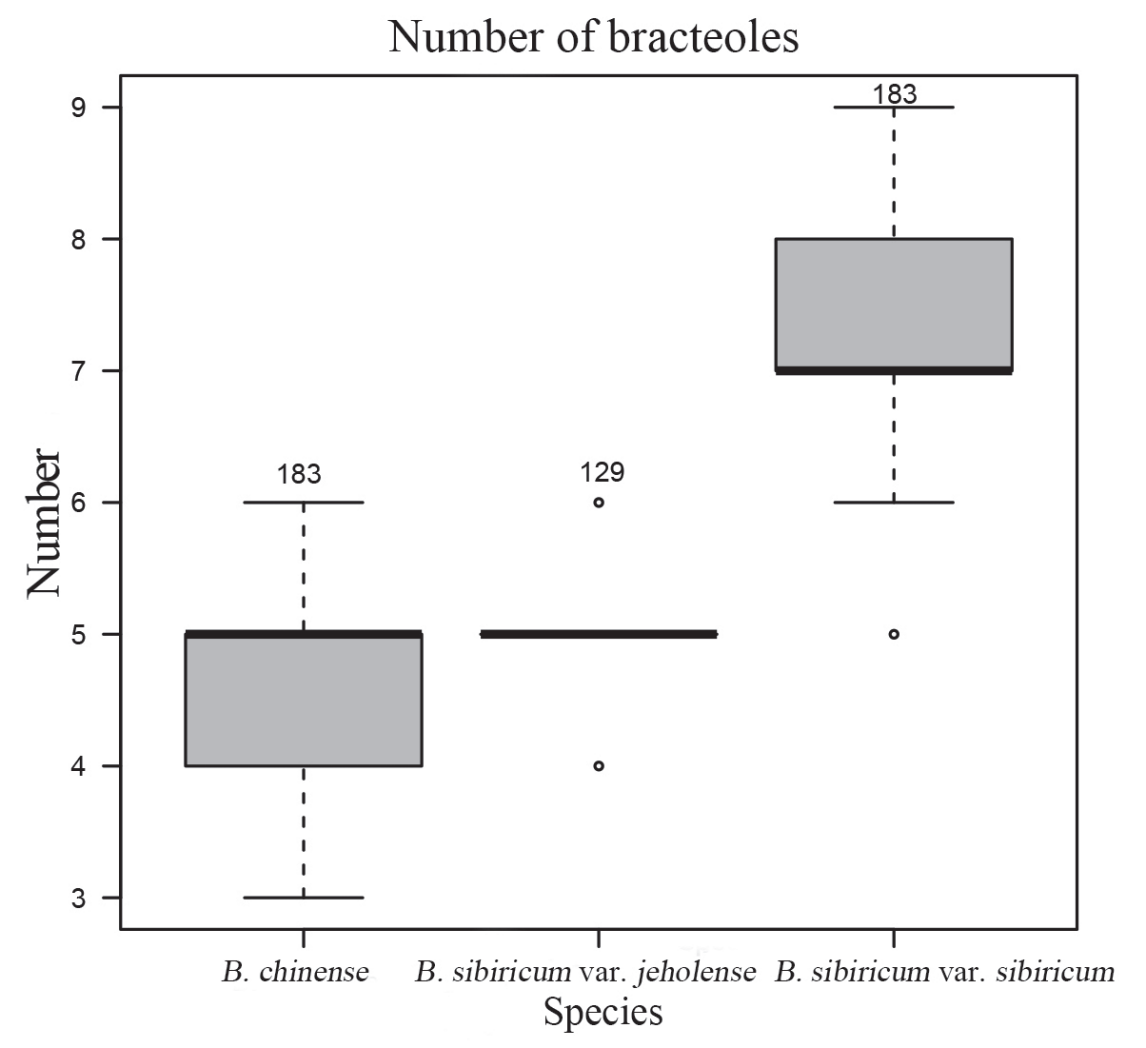
Comparison of the number of bracteoles found in *Bupleurumchinense*, B.sibiricumvar.jeholense and B.sibiricumvar.sibiricum.

**Figure 2.**
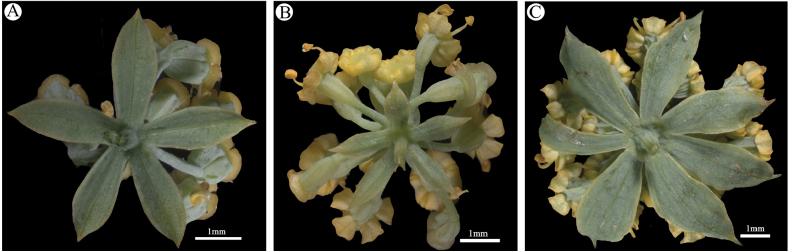
Umbel and bracteoles of the taxa under study **A***Bupleurumchinense***B**B.sibiricumvar.jeholense**C**B.sibiricumvar.sibiricum.

**Figure 3.**
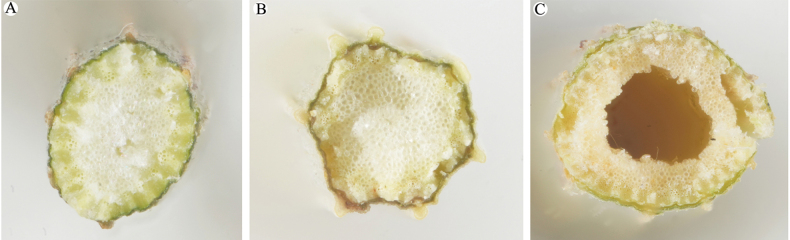
Stem transections of the taxa under study **A***Bupleurumchinense***B**B.sibiricumvar.jeholense**C**B.sibiricumvar.sibiricum.

The bean plot indicated that the number of bracteoles of B.sibiricumvar.jeholense was mostly 5 and occasionally 6, whereas that of B.sibiricumvar.sibiricum was (6)7–8(9). *Bupleurumchinense* had 5, sometimes 4 or even 3 bracteoles at lower elevations or in an understory (Figs 1, 2). The stems of *B.chinense* and B.sibiricumvar.jeholense were solid, lacking a cavity, whereas those of B.sibiricumvar.sibiricum were hollow at all internodes, which led to the formation of a cavity (Fig. 3). Bupleurumsibiricumvar.jeholense and *B.chinense* were found to mainly differ in height, number of stems, and presence of middle stem leaves. The morphological characteristics of B.sibiricumvar.jeholense plants are as follows: height below 40 cm, several stems, with 1–2 branches per stem, middle stem leaves narrower, and middle leaf length to width ratio 10–16. In contrast, *B.chinense* plants had single, occasionally several stem 40–90 cm high, with 2–4 branches per stem, and middle stem leaf length to width ratio 6–10. A comparison of morphological characters is shown in Table 2.

**Table 2.** Comparison of the morphological characteristics and geographic distributions of three taxa under study.

Character	*B.chinense*	B.sibiricumvar.jeholense	B.sibiricumvar.sibiricum
**Height**	40–90 cm	20–40 cm	30–70 cm
**Stem (number)**	Single, occasionally several	Many, clustered	Many, clustered
**Stem (branching)**	2–4-branched	1–2-branched	1–2-branched
**Stem (presence of cavity)**	Solid	Solid	Hollow in all internodes
**Basal leaves**	Withering early	Withering early	Persistent, many
**Basal leaf size**	4–7 × 0.6–0.8 cm	5–10 × 0.3–0.8 cm	12–25 × 0.7–1.6 cm
**Middle stem leaves**	4–12 × 0.6–1.8 cm	6–12 × 0.5–1.2 cm	6–14 × 0.5–1.6 cm
**Middle stem leaf length to width ratio**	6–10	10–16	10–14
**Upper stem leaves**	Not embracing	Not embracing	Rounded-cuneate, embracing
**Bracteole relative length**	Shorter than flowers	Shorter than or equal to flowers	Exceeding flowers
**Bracteoles**	(3–4) 5, lanceolate	5 (6–7), lanceolate	(6) 7–12, elliptic-lanceolate
**Distribution**	Northeast and Central China	Yan mountains (Hebei and Beijing)	Heilongjiang, Liaoning, Inner Mongolia, Mongolia, and Russia

### ﻿Chromosome numbers

**Figure 4.**
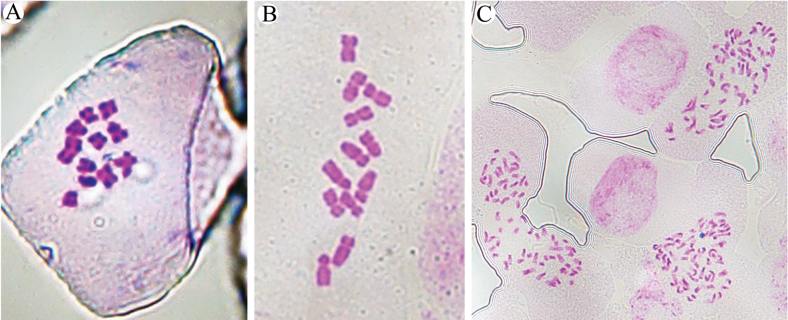
Metaphase chromosomes of the taxa under study **A***Bupleurumchinense***B**B.sibiricumvar.jeholense**C**B.sibiricumvar.sibiricum.

Cytological analysis revealed that the chromosome number of B.sibiricumvar.jeholense was 2n = 12 (x = 6), i.e., the same as *B.chinense* but different from B.sibiricumvar.sibiricum (2n = 64, x = 8) (Fig. 4). We report the chromosome number of B.sibiricumvar.jeholense for the first time, and the chromosome numbers of *B.chinense* and B.sibiricumvar.sibiricum determined here are consistent with previous reports (Pan et al. 1995; Qin et al. 1989).

### ﻿Phylogenetic analysis

**Figure 5.**
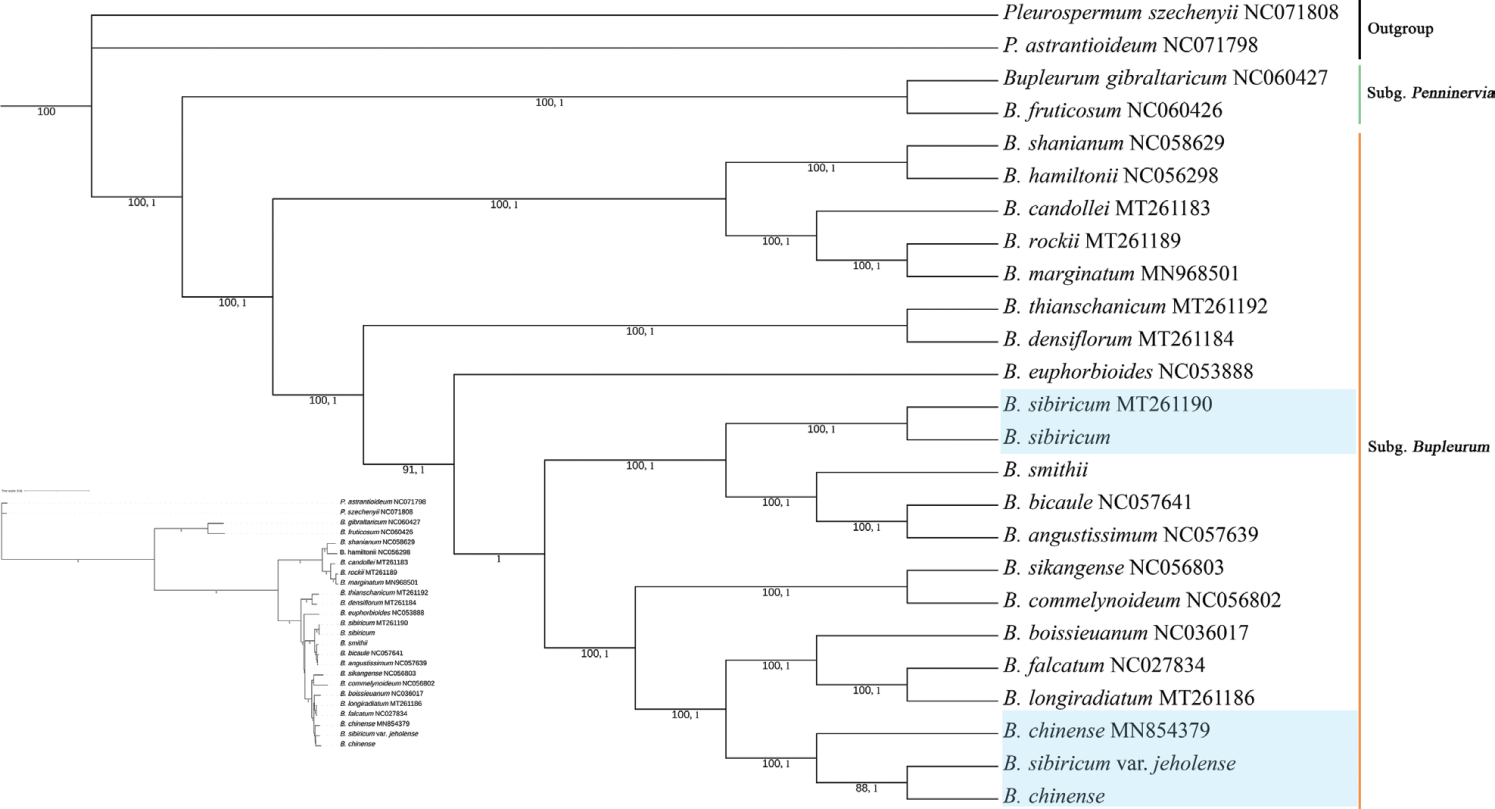
Phylogenetic tree of some Chinese *Bupleurum* species as inferred from chloroplast genomes using ML analyses (numbers below branches represent ML bootstrap values and BI posterior probabilities).

The size of the four *Bupleurum* chloroplast genomes ranged from 155,706 to 155,858 bp. The complete chloroplast genome had a typical circular quadripartite structure and consisted of a pair of inverted repeat regions separated by the large single copy and small single copy regions. The topologies of the ML and BI trees constructed using the cp genome sequences were consistent. These results showed that the genus *Bupleurum* can be divided into two clades, with all Chinese *Bupleurum* plants belonging to B.subg.Bupleurum. Bupleurumsibiricumvar.sibiricum was found to cluster with *B.smithii* and *B.bicaule* Helm (BS = 100% PP = 1), whereas *B.chinense* was found to cluster with B.sibiricumvar.jeholense (BS = 100% PP = 1) and was more distantly related to *B.longiradiatum* Turcz., *B.falcatum* L. and *B.boissieuanum* H. Wolff (Fig. 5).

## ﻿Discussion

### ﻿Comparative morphology

Botanists have closely monitored the number of bracteoles because this character is essential in the taxonomy of *Bupleurum*. Moreover, bracteole number is an important indicator of the taxonomic position of B.sibiricumvar.jeholense in the genus. Five bracteoles are generally present in both *B.chinense* and B.sibiricumvar.jeholense. However, in environments such as the understory, *B.chinense* may have only three or four bracteoles. Furthermore, 6–7 bracteoles have occasionally been observed in B.sibiricumvar.jeholense. We observed that the number of bracteoles in *B.sibiricum* samples collected from the Daqing Mountain, which were collected at the same latitude and altitude as the samples collected from Wuling Mountain, was not 5, as would be expected for B.sibiricumvar.jeholense. Thus, we speculated that the 5-bracteoled B.sibiricumvar.jeholense may not have evolved from the 12-bracteoled B.sibiricumvar.sibiricum.

In the classification of the genus *Bupleurum*, stem structural characteristics have rarely been examined. After observing numerous specimens, we found that the stem of B.sibiricumvar.sibiricum was hollow and contained a substantial cavity. In contrast, the stems of *B.chinense* and B.sibiricumvar.jeholense lacked this cavity. The presence or absence of a stem cavity is a stable character and does not change with the period of growth. For example, *B.komarovianum* was once treated as a variety of *B.chinense* (Liaoning Forestry Soil Research Institute 1977) until Wang et al. (2011b) found that the stems of *B.komarovianum* were hollow. The authors combined morphological and chromosomal evidence to argue that *B.komarovianum* should be reinstated as a separate species. Finally, the basal leaves of B.sibiricumvar.sibiricum were found to be persistent, whereas those of B.sibiricumvar.jeholense were found to wither at the flowering and fruiting stages, as in *B.chinense*.

*Bupleurumchinense* is widely distributed throughout East Asia and is often cultivated as a medicinal plant. The morphology of this species varies with the environment. Bupleurumsibiricumvar.jeholense may be a variant of *B.chinense* that has adapted to the cold environments found in high-altitude mountains. In particular, the bracteole number may have increased to protect flowers at higher altitudes (Kofidis et al. 2007). Compared with *B.chinense* plants, B.sibiricumvar.jeholense plants are shorter, with several stems, fewer stem branches, and narrower middle stem leaves. Overall, the above evidence suggests that B.sibiricumvar.jeholense should be treated as a variety of *B.chinense*.

### ﻿Cytological analysis

Chromosomal variation plays a vital role in species formation, and the diversity of chromosome size and number is therefore an important character that can be used to track *Bupleurum* species that have adapted to different habitats (Wang 2011a; Weiss-Schneeweiss and Schneeweiss 2013). Bupleurumsibiricumvar.jeholense differs from B.sibiricumvar.sibiricum in both chromosome number and basal number, but these values are identical to those of *B.chinense*. In this study, *B.chinense* and B.sibiricumvar.jeholense were both found to have a chromosome number of 12 (diploid), which is the common basic number reported for this genus (Wang 2011a). Regarding the reported chromosome number of 64 in *B.sibiricum* from a population collected in Inner Mongolia, Qin et al. (1989) and Jiang et al. (2002) assumed that *B.sibiricum* was octoploid (i.e., with a basal number of 8), marking the highest level of polyploidy found in this genus. This observation may be due to chromosomal polyploidy, a type common in colder climates (Jiang et al. 2002). Thus, in terms of both chromosome number and basal number, B.sibiricumvar.jeholense appears to be only distantly related to B.sibiricumvar.sibiricum.

### ﻿Phylogenetic analysis

In this study, a phylogenetic tree was reconstructed using chloroplast genome data. Our findings were consistent with those of Wang et al. (2011b). In particular, we found that Bupleurumsibiricumvar.jeholense was embedded in *B.chinense*, which forms a sister clade with *B.yinchowense*. In addition, B.sibiricumvar.sibiricum is sister to *B.smithii* and *B.bicaule*. In contrast to *B.chinense*, the distributions of *B.sibiricum* and *B.bicaule* are ranging from Siberia to northeastern China.

### ﻿Distribution

*Bupleurumsibiricum* is distributed widely throughout temperate Asia. It often co-occurs with *B.scorzonerifolium* in arid meadows in Inner Mongolia and Siberia at elevations of 700–2000 m. In the field, B.sibiricumvar.jeholense is often found to co-occur with *B.chinense* at different altitudes in the same mountain. At present, B.sibiricumvar.jeholense is found only in high-altitude areas of the Yan Mountains. Moreover, it has a narrow distribution area and is confined to altitudes of 1500–2000 m. In contrast, *B.chinense* is widely distributed and is found at altitudes ranging from 200 to 1600 m in Northeast China; however, it has also been found isolated at an altitude of 2100 m in Northwest China. Given these findings, we speculate that B.sibiricumvar.jeholense is a specialized morphological variant of *B.chinense* that has specifically adapted to high altitudes. Overall, in terms of distribution and habitat, B.sibiricumvar.jeholense differs considerably from *B.sibiricum* and is more similar to *B.chinense*.

### ﻿Taxonomic treatment

#### ﻿ Bupleurum chinense var. jeholense

Taxon classificationPlantaeApialesApiaceae

(Nakai) Q.R.Liu & L.H.Wang comb. nov.

3EBB3207-7F5B-5758-B228-F9C187CC216E

urn:lsid:ipni.org:names:77335467-1

– Bupleurumjeholense Nakai in J. Jap. Bot. 13: 482 (1937) – Bupleurumsibiricumvar.jeholense (Nakai) Chu in Shan & Li, Acta Phytotax. Sin. 12 (3): 272 (1974).
= Bupleurumjeholensevar.latifolium Nakai in J. Jap. Bot. 13: 482 (1937). Type. China. Hebei: Wuling Mountain, 1800 m, 2 Sept 1933, *Nakai*, *Honda et Kitagawa s. n.* (holotype: TI0082957!).

##### Type.

**Figure 6.**
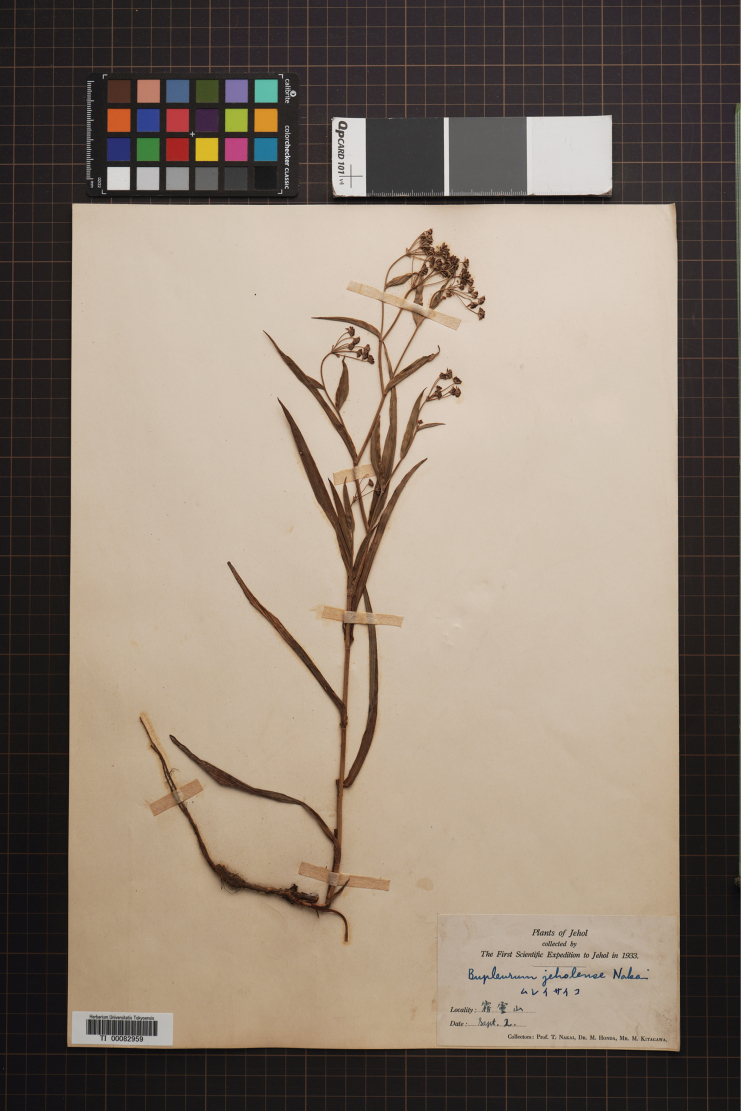
Isotype of Bupleurumchinensevar.jeholense (Photo from TI Herbarium).

China. Hebei: Wuling Mountain, 1500–2000 m, 2 Sept 1933, *Nakai*, *Honda et Kitagawa*, *s. n.* (holotype: not barcode, fig in protologue pp 481!; isotypes: TI0082958! TI0082959! (Fig. 6) TI0082960! TI0082961!],

##### Diagnosis.

Bupleurumchinensevar.jeholense is morphologically similar to var. chinense and can be distinguished from the latter by plant size (>40 cm), the presence of multiple stems, with 1–2 branches per stem, thinner middle stem leaves, leaf length to width ratio 10–16, and the presence of 5 bracteoles.

##### Description.

**Figure 7.**
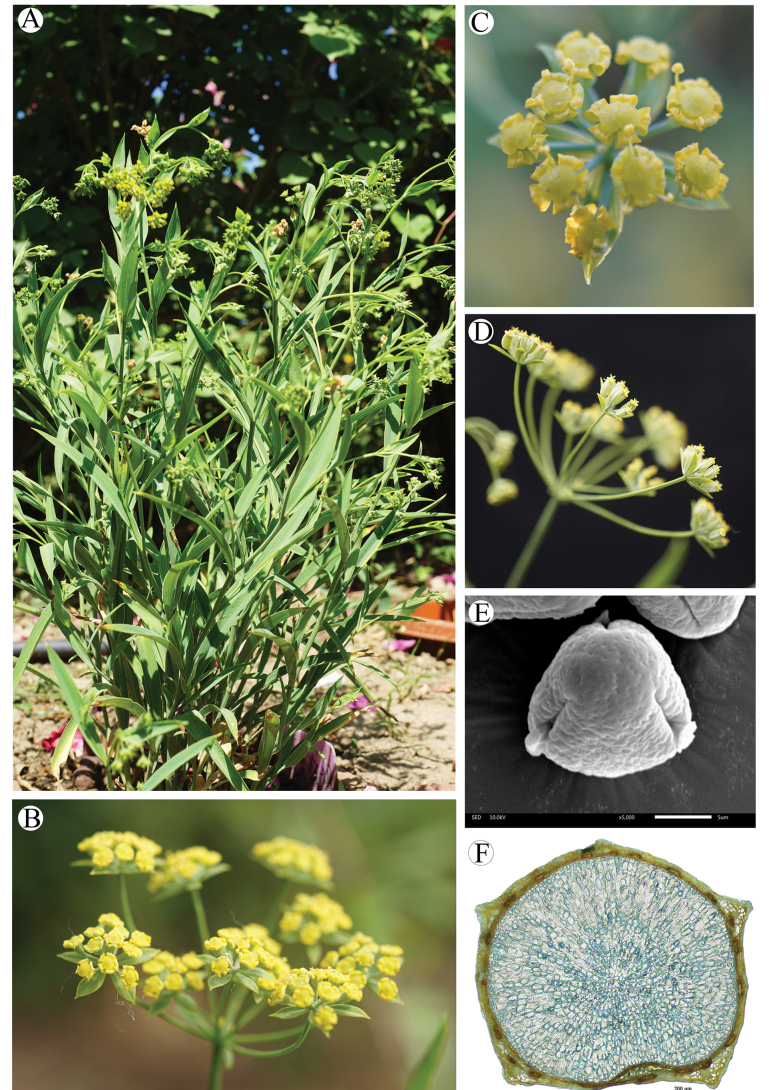
Bupleurumchinensevar.jeholense**A** plant **B** umbel and bract **C** flowers **D** umbel (side view) **E** pollen **F** transverse section of mericarp.

Plants 20–40 cm, perennial. Root stout, brown, woody. Stem solitary or several, solid, petioles often purplish-red, clasping base without fibrous remnant sheaths. Basal leaves oblanceolate, 5–10 × 0.3–0.8 cm, base petioles, apex acuminate. Middle leaves sessile, oblanceolate, 4.5–12 × 0.4–1.4 cm, 7–9-nerved, base tapering, apex obtuse or acute, apiculate. Apical leaves small. Umbels 5–12, nearly equal or unequal rays 0.4–4 cm long; bracts of 1–5 unequal leaflets, often obsolete or deciduous, 3–15 × 0.6–11 mm, 4–7-nerved; bracteoles 5, lanceolate, 3–7 × 0.6–0.8 mm, exceeding flowers; umbellule 4–12 mm across, 8–14-flowered. Petals bright yellow. Stylopodium low-conic, discoid, dark yellow. Fruit oblong, brown, ca. 2.2–3.5 × 0.9–1.5 mm; ribs prominent, narrowly winged, wings pale brown; vittae 3(–4) in each furrow, 4 on commissure (Fig. 7). Fl. July–August and Fr. August–October. 2n = 12.

##### Phenology.

Flowering and fruiting from August to October.

##### Distribution and habit.

Hebei, Beijing. It grows in mountains at elevations of 1500–2000 m.

##### Additional specimens examined.

China. **Beijing**: Fangshan county, *Jin-Wu Wang s.n.* (PEY!); Mentougou county, *Xiao-Liu QS-186* (BJFC!); *Quan-Ru Liu 200609009* (BNU!); *Xue Lin 05 05-4-114* (BJFC!); *Gang-Min Zhang 201008036* (BJFC!); *Xian-Yun Mu 1821* (BJFC!); *Duan-Zheng Lu s.n.* (BJFC!); *Quan-Ru Liu DL025-2* (BNU!); *Quan-Ru Liu DL026-2* (BNU!); *Quan-Ru Liu DL027-1* (BNU!); Miyun county, *Xian-Yun Mu 1924* (BJFC!). **Hebei**: Xinglong county, *Li-Hua Wang BNU2021HB029* (BNU!); *Li-Hua Wang BNU2021HB025* (BNU!); *Jia-Yi Liu 0845 2190* (TIE!); *Jin-Wu Wang s.n.* (PEY!); *Zhen-Fu Fang 825* (NAS!); *Ze-Hui Pan 83940* (NAS!); *Ze-Hui Pan 83939* (NAS!); *Shen-E Liu 4833* (IFP!); *Zhen-Fu Fang 826* (IFP!); *Quan-Ru Liu WLS068* (BNU!); *Xin-Yuan Liu 1652* (KUN!); *Jia-Yi Liu 08450* (TIE!); *Jia-Yi Liu 00485* (TIE!); *Biaobenshi 2190* (PE!); *Wu-Xiu Zhang 91* (PE!).

## Supplementary Material

XML Treatment for Bupleurum chinense var. jeholense

## ﻿Additional information

### Author ORCIDs

Yi He https://orcid.org/0000-0002-6925-7299

Quan-Ru Liu https://orcid.org/0000-0003-4270-4746

### Data availability

All of the data that support the findings of this study are available in the main text.
